# GLI Transcriptional Targets S100A7 and KRT16 Show Upregulated Expression Patterns in Epidermis Overlying the Tumor Mass in Melanoma Samples

**DOI:** 10.3390/ijms25116084

**Published:** 2024-05-31

**Authors:** Matea Kurtović, Nikolina Piteša, Josipa Čonkaš, Helena Hajpek, Majda Vučić, Vesna Musani, Petar Ozretić, Maja Sabol

**Affiliations:** 1 Division of Molecular Medicine, Ruđer Bošković Institute, Bijenička cesta 54, 10000 Zagreb, Croatia; matea.kurtovic@irb.hr (M.K.); nikolina.pitesa@irb.hr (N.P.); josipa.conkas@irb.hr (J.Č.); hhajpek@stud.biol.pmf.hr (H.H.); vmusani@irb.hr (V.M.); pozretic@irb.hr (P.O.); 2 Department of Biology, Faculty of Science, University of Zagreb, Horvatovac 102a, 10000 Zagreb, Croatia; 3 Ljudevit Jurak Clinical Department of Pathology and Cytology, Sestre Milosrdnice University Hospital Center, 10000 Zagreb, Croatia; majda.vucic@kbcsm.hr; 4 Department of Pathology, School of Dental Medicine, University of Zagreb, 10000 Zagreb, Croatia

**Keywords:** melanoma, HH-GLI, biomarkers, keratins, epidermis, S100A7

## Abstract

Although not completely understood, the role of the Hedgehog-GLI (HH-GLI) signaling pathway in melanoma and epithelial skin tumors has been reported before. In this study, we confirmed in various melanoma cell line models that keratin 16 (KRT16) and S100 Calcium-Binding Protein A7 (S100A7) are transcriptional targets of GLI Family Zinc Finger (GLI) proteins. Besides their important role in protecting and maintaining the epidermal barrier, keratins are somehow tightly connected with the S100 family of proteins. We found that stronger expression of KRT16 indeed corresponds to stronger expression of S100A7 in our clinical melanoma samples. We also report a trend regarding staining of GLI1, which corresponds to stronger staining of GLI3, KRT16, and S100A7 proteins. The most interesting of our findings is that all the proteins are detected specifically in the epidermis overlying the tumor, but rarely in the tumor itself. The examined proteins were also not detected in the healthy epidermis at the edges of the sample, suggesting that the staining is specific to the epidermis overlaying the tumor mass. Of all proteins, only S100A7 demonstrated a statistically significant trend regarding tumor staging and staining intensity. Results from our clinical samples prove that immune infiltration is an important feature of melanoma. Pigmentophages and tumor-infiltrating lymphocytes (TIL) demonstrate a significant association with tumor stage, while mononuclear cells are equally present in all stages. For S100A7, we found an association between the number of TILs and staining intensity. Considering these new findings presented in our study, we suggest a more detailed examination of the possible role of the S100A7 protein as a biomarker in melanoma.

## 1. Introduction

Melanoma is a very aggressive type of cancer that arises from melanocytes, and it usually occurs with a high incidence in people who are positive for the BRAFV600E or NRASQ61R mutation [1]. The Hedgehog-GLI (HH-GLI) signaling pathway is a developmental pathway that is, among many other processes, involved in the differentiation of the skin during embryogenesis. In the skin of adult organisms, the expression of the HH-GLI pathway components is found in hair follicles but not detected in epidermal keratinocytes [2]. Although not completely understood, the role of the HH-GLI signaling pathway in melanoma and epithelial skin tumors has been reported before. For example, significantly increased expression of GLI1 and GLI2 has been reported in basal cell carcinoma [3,4]. Also, inactivating mutations in PTCH1 occur in about 70–80% of basocellular carcinomas [5]. It has been shown that the HH-GLI pathway is required for the normal proliferation of human melanocytes in vitro and that GLI1 and PTCH1 are upregulated in skin metastases compared to primary melanomas [6]. Additionally, melanoma growth in vitro and in vivo is reduced by the HH-GLI pathway inhibitor, sonidegib [7], and increased levels of signaling pathway components correlate with poor survival in melanoma patients [8]. Even though the activity of the HH-GLI pathway in adult organisms is attenuated, it can be triggered by various environmental factors, including UV exposure and wounding [9]. One of the identified important factors affecting melanoma development is tumor–stroma communication. The release of various growth factors and cytokines from the surrounding tissues promotes tumor growth. This is also true for the role of HH-GLI signaling in melanoma [10,11]. In our previous research [12], we have identified novel targets of GLI proteins in melanoma using RNA sequencing. Among the 15 targets that were successfully validated on a panel of melanoma cell lines, two of them, namely, S100A7 and KRT16, have emerged as particularly interesting. 

The involvement of S100 proteins in melanoma progression is well known, and different family members may have different roles. For example, S100B is a known marker of melanoma, and some other members of the S100A family, like S100A2 and S100A6, are thought to have a role in the progression of melanoma from normal melanocytes to metastatic disease [13]. S100A7 (also known as psoriasin) is a member of the S100 family of calcium-binding proteins that was first identified as a 11.4 kDa cytoplasmic and secreted protein isolated from skin affected by psoriasis [14]. One study points out that upregulation of S100A7 is not restricted only to psoriasis; strong S100A7 expression was also found in atopic dermatitis, mycosis fungoides, Darier’s disease, and lichen sclerosus et atrophicus. A study suggested that common pathological features of these inflammatory diseases lead to the expression of S100A7 [15]. All 25 members of the S100 protein family are Ca^2+^-responsive proteins, which dimerize upon calcium binding and regulate various intracellular and extracellular processes, such as cell proliferation, differentiation, migration, invasion, apoptosis, energy metabolism, and inflammation [16]. Cultured fibroblasts do not express S100A7, and it is rarely detected in the normal skin and normal melanocytes [17,18]. Keratinocytes, on the other hand, express S100A7, and its expression can be additionally stimulated by extracellular calcium levels, anoikis, cell confluency [19], oxidative stress, hypoxia [20], and microbial infection [21]. Expression of S100A7, like the expression of the abovementioned components of the HH-GLI pathway, can also be induced by exposure to UV light both in vitro and in vivo [22,23] and by skin injury, where it is upregulated at the wound edges, promotes wound closure, and acts as an antimicrobial peptide [24,25,26]. Many members of the S100 family of proteins are involved in the host defense mechanism, most notably S100A8/9. S100A7 can act as a chemotactic signal for the cells of the immune system; it can trigger the synthesis of cytokines and chemokines; and it is involved in maintaining the skin barrier [27]. It has been detected in the skin of newborns at birth, where it may play a role in protection from microbial infection at birth [28]. Additionally, S100A7 has direct bactericidal activity against *E. coli* [21].

The intermediate filaments that make up the cytoskeleton of the mammalian epithelium are most often composed of type I and type II keratins. These two large families comprise a total of 56 genes. Besides their known role as providers of structural support to epithelial cells, evidence has shown that keratins also regulate cell proliferation, migration, adhesion, and inflammatory features of keratinocytes [29]. KRT16 is a type I keratin and a known regulator of innate immunity in the skin. Interestingly, one study from Bhawan et al. [30] reports subtle staining of KRT16 in epidermal melanocytes of normal human skin. However, a study by Ramot et al. wished to confirm or refute the expression of KRT16 in normal human epidermal and/or hair follicle melanocytes in situ, and their results indicate that human epidermal or hair follicle melanocytes do not express KRT16 [31]. KRT16 is also found to be significantly downregulated in metastatic melanoma. In a study by Metri et al., it was determined that its expression is the biggest discriminating factor between primary and metastatic melanoma [32]. KRT16 is also involved in the immune response, as it controls the response to injury and damage signals. Investigations on a mouse model have shown that KRT16 regulates an important inflammatory checkpoint, and its loss results in hyperactivation of the immune response to the skin [33]. 

Taken together, evidence shows that S100A7 and KRT16 could be interesting targets of GLI proteins to investigate in vitro and ex vivo. Therefore, we have decided to examine the association of GLI proteins and their targets, S100A7 and KRT16, in melanoma tissues and investigate their association with the level of immune cell infiltration in melanoma.

## 2. Results

### 2.1. S100A7 and KRT16 Are Novel Targets of GLI1 in Different In Vitro Models of Melanoma Cell Lines

**Figure 1:**
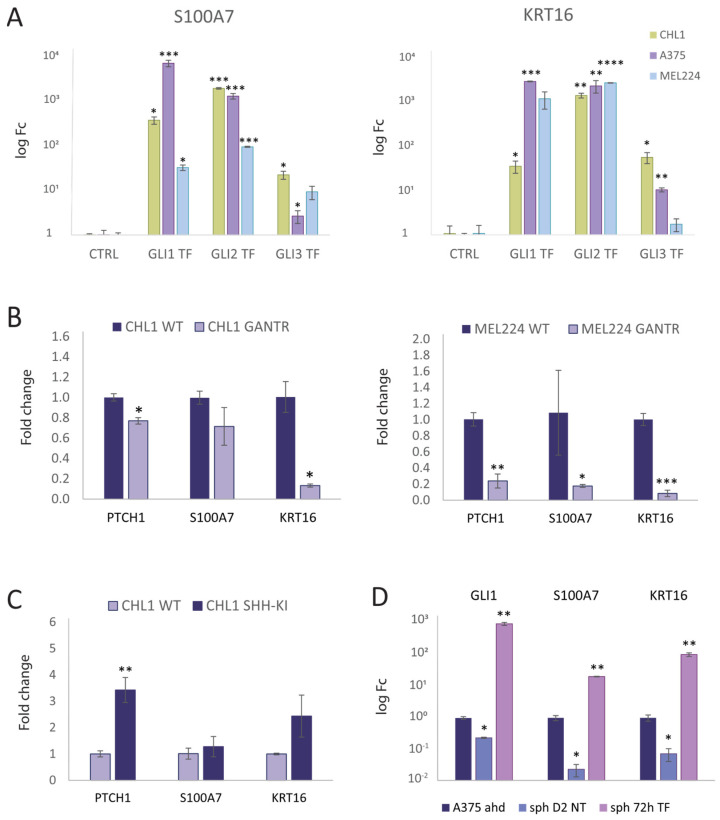
(**A**) Expression of S100A7 and KRT16 in three melanoma cell lines (CHL-1, A375, and MEL224), after transfection (TF) with GLI1, GLI2, and GLI3 expression plasmids. (**B**) Validation of *S100A7*, *KRT16*, and a known target of GLI proteins, *PTCH1*, in cell lines CHL-1 and MEL224 resistant to inhibitor GANT61 (GANTR), a model for pathway downregulation. (**C**) Validation of *S100A7* and *KRT16* gene expression in the CHL-1 cell line with stable overexpression of SHH, a model of autocrine pathway upregulation. The relative gene expression of *S100A7* and *KRT16*, as well as the *PTCH1* gene, is increased, but not statistically significant for *S100A7* and *KRT16*. (**D**) Validation of *S100A7* and *KRT16* gene expression in spheroid culture (sph) of the A375 cell line. Dark blue columns show adherent A375 cell line (adh), light purple columns show non-transfected spheroids of A375 cell line, and pink columns show spheroids transfected with GLI1 after 72 h. Data are presented with mean value ± standard deviation (SD), *, *p* < 0.05; **, *p* < 0.01; ***, *p* < 0.001; ****, *p* < 0.0001.

Using the UCSC Genome Browser database, we found that promotor regions of both KRT16 and S100A7 genes contain binding sites for GLI1, GLI2, and GLI3 transcription factors [34]. To investigate if S100A7 and KRT16 are indeed transcriptionally regulated by GLI proteins, three melanoma cell lines were transfected with the GLI1, GLI2, or GLI3 expression plasmid, and the expression of *S100A7* and *KRT16* was measured by qPCR. Overexpression of all three GLI proteins upregulates the expression of two target genes in all cell lines, but upregulation by GLI1 or GLI2 is the most statistically significant (Figure 1A). For the model of HH-GLI signaling downregulation, we used cell lines resistant to the inhibitor GANT61, for which we have previously demonstrated that the Hedgehog signaling pathway is downregulated [35]. Figure 1B shows the expression analysis of target genes *PTCH1*, *S100A7*, and *KRT16* in these lines. Expression of *PTCH1* was used as a positive control. In the cell line MEL224 resistant to GANT61 (MEL224 GANTR), the expressions of all three target genes are statistically significantly reduced. This effect is also visible in the cell line CHL-1 resistant to GANT61 (CHL-1 GANTR), but it is not statistically significant for *S100A7*. As a model for autocrine upregulation of the pathway, a cell line with a stable knock-in of SHH was used (CHL-1 SHH KI). Figure 1C shows that in cells stably overexpressing SHH, the relative gene expression of *S100A7* and *KRT16*, as well as the *PTCH1* gene, is increased, but not statistically significant for *S100A7* and *KRT16*. Finally, to investigate the changes in gene expression in 3D conditions of tumor cells, spheroids from the A375 line were generated using the hanging drop method. The expression of *GLI1* is downregulated in spheroids compared to the adherent cell line, and this is followed by the downregulation of *S100A7* and *KRT16*. If the cells transfected with GLI1 are used to generate spheroids, then the upregulation of *GLI1* is followed by the upregulation of both *S100A7* and *KRT16* (Figure 1D). All these data taken together strongly suggest that both *S100A7* and *KRT16* expressions are transcriptionally regulated by GLI1 in melanoma cell lines.

### 2.2. Clinical Characteristics of Melanoma Samples Are Correlated with Stages of the Disease

**Figure 2:**
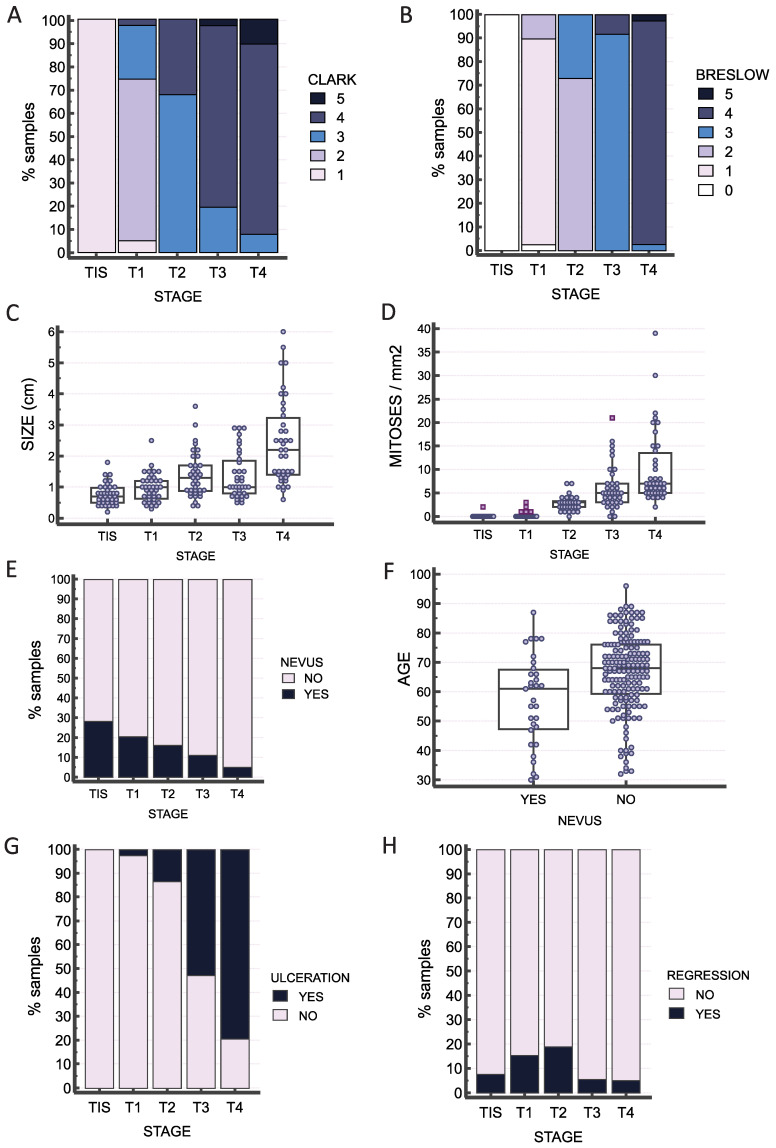
Association of clinical characteristics with tumor stage in the sample set. Stage is associated with (**A**) Clark (*p* < 0.0001), (**B**) Breslow (*p* < 0.0001), (**C**) tumor size (*p* = 0.003), and (**D**) number of mitoses/mm^2^ (*p* < 0.0001). (**E**) Association between stage and the presence of preexisting nevus (*p* = 0.003). (**F**) Melanoma within a preexisting nevus is detected at a slightly earlier age than melanoma de novo (*p* = 0.005). (**G**) Ulceration increases with the tumor stage (*p* < 0.0001), and (**H**) regression is most frequently detected in T2 samples but without statistically significant differences between stages (*p* = 0.183).

For the purpose of examining the expression of GLI proteins and their targets in melanoma, 190 samples of FFPE melanoma tissues were collected. As expected, the stage, Clark and Breslow parameters, tumor size, and number of mitoses were associated, confirmed with the Chi-square tests (Figure 2A–D, Supplementary Table S1). As some of the melanoma samples showed evidence of preexisting nevus (31/190, 16.31%), we decided to analyze if there were any differences between the melanoma that developed de novo and the ones that developed from a preexisting nevus. The highest proportion of preexisting nevus was found in the lowest stages (28.2% of TIS samples vs. 5.1% of T4 samples) (*p* = 0.003), as shown in Figure 2E. There is also a significant difference in the occurrence of melanoma within the preexisting nevus according to age (*p* = 0.047), with nevus-associated melanoma being detected/diagnosed at a slightly younger age (Figure 2F). Ulceration increases with the tumor stage (*p* < 0.0001), as shown in Figure 2G. Regression was observed in a total of 20 samples (10.52%), with the most samples with regression in T2 samples, even though their differences were not statistically significant (Figure 2H). This may be due to the small number of samples with regression within the whole set.

### 2.3. Proteins S100A7 and KRT16 Have Strong Expression in the Epidermis Overlying the Tumor

**Figure 3:**
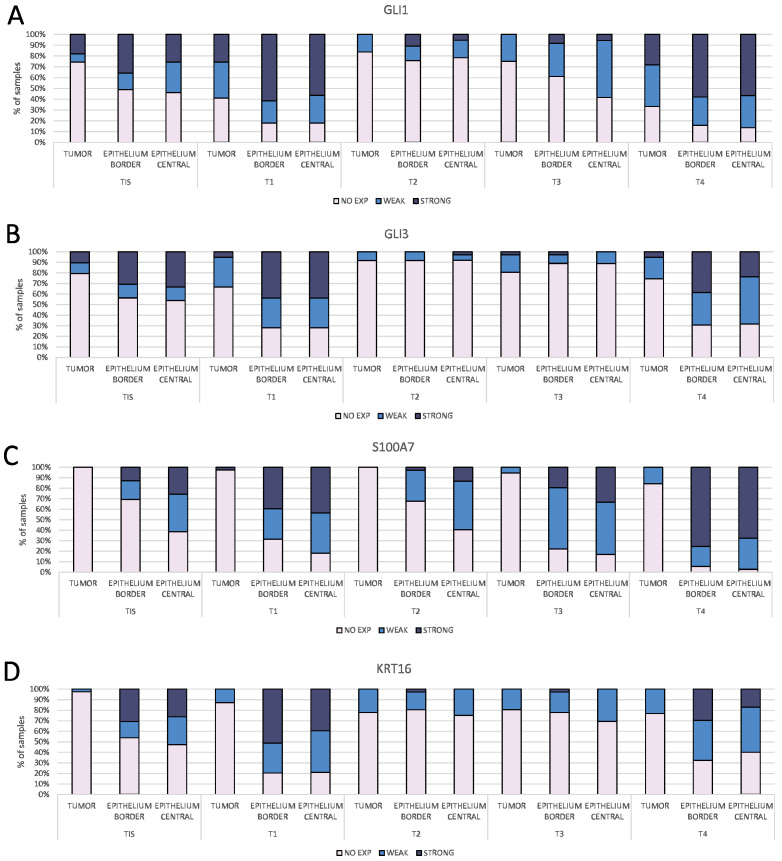
Proportion of samples with staining intensities (no expression, weak or strong) for (**A**) GLI1, (**B**) GLI3, (**C**) KRT16, and (**D**) S100A7 in different melanoma stages (TIS-T4). For each sample, the intensity was measured at three locations: tumor mass (TUMOR), epidermis bordering the tumor mass (BORDER), and epidermis overlying the tumor mass (CENTRAL). In general, the staining intensities for all proteins were stronger in the epidermis (both border and central) compared to the tumor mass.

GLI1, GLI3, S100A7, and KRT16 staining were successfully detected in our samples, but GLI2 was undetectable in all stained slides and was not analyzed further. Interestingly, most of the positive staining was detected in the epidermis overlying or bordering the tumor mass, while the tumor mass had strong staining only in a smaller proportion of samples for GLI1 and GLI3 (Figure 3, Supplementary Table S1). 

**Figure 4:**
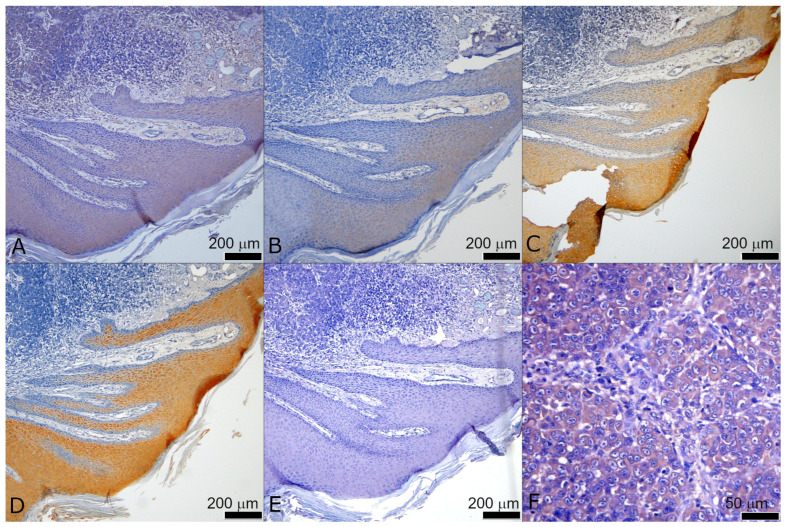
Representative staining of the border area of the T4 tumor for (**A**) GLI1, (**B**) GLI3, (**C**) KRT16, and (**D**) S100A7. (**E**) Negative control (no primary antibody), and (**F**) higher magnification of GLI1 staining in the tumor. The scale bar for (**A**–**E**) was 200 μm and for (**F**) was 50 μm.

**Figure 5:**
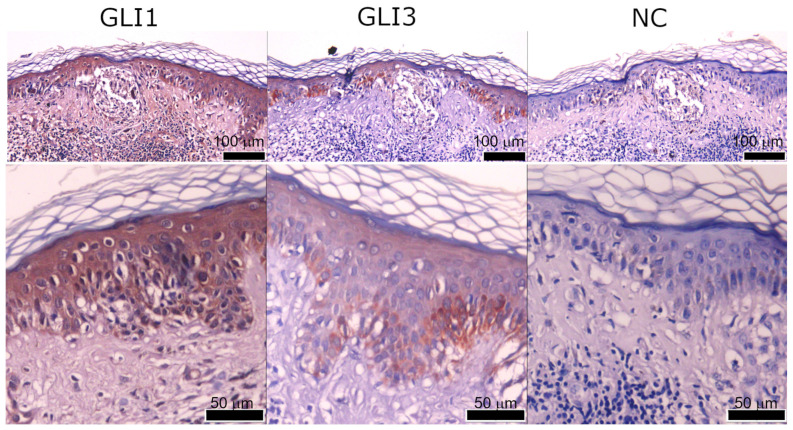
Different localizations of GLI staining. GLI3 shows stronger staining in the basal layer in the epidermis of low-stage melanoma, while GLI1 intensity is uniform through the epidermis overlying the tumor. NC denotes negative control. The scale bar for the upper panel was 100 μm, and for the lower panel, it was 50 μm.

Expression of S100A7 and KRT16 was rarely detected in the tumor mass (Figure 3, Figure 4 and Supplementary Figures S1–S3). All four proteins show staining in the same structures in the epidermis of high-stage tumors (Figure 4), but in low-stage tumors, GLI3 often shows stronger staining in the basal cell layer (Supplementary Figure S2). Examined proteins were not detected in the healthy epidermis at the edges of the sample (where available), suggesting that the staining is specific for the epidermis overlaying or bordering the tumor (Figure 5). 

### 2.4. Expression of GLI1 Is Strongly Associated with Expressions of GLI3, KRT16, and S100A7 in the Epidermis Bordering the Tumor

**Figure 6:**
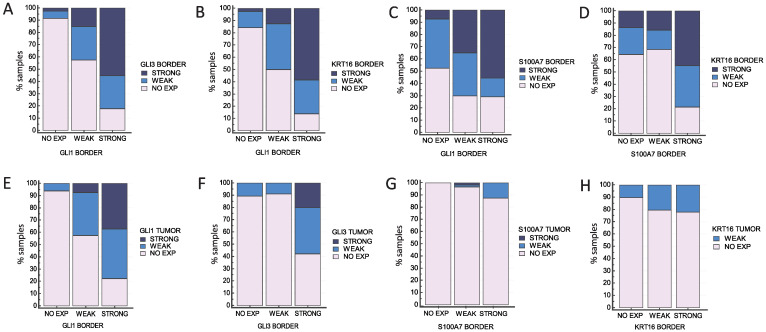
Comparison of protein expressions (**A**) GLI1 vs. GLI3, (**B**) GLI1 vs. KRT16, (**C**) GLI1 vs. S100A7, and (**D**) S100A7 vs. KRT16 in the same location (epidermis bordering the tumor = BORDER). There is a significant association between GLI1 expression and expressions of all other tested proteins for border vs. border (*p* < 0.0001). (**E**–**H**) Expression of the same proteins in BORDER vs. tumor mass (TUMOR). GLI1 (**E**), GLI3 (**F**), and S100A7 expression (**G**) in the tumor tissue is significantly associated with the expression in the border epidermis (*p* < 0.0001 for all three proteins), while KRT16 expression (**H**) is not significantly associated (*p* = 0.107).

The association between the two locations in the epidermal layer is significant for the tested proteins: GLI1 border vs. GLI1 central (*p* < 0.0001), GLI3 border vs. GLI3 central (*p* < 0.0001), KRT16 border vs. KRT16 central (*p* < 0.0001), and S100A7 border vs. S100A7 central (*p* < 0.0001). In general, it seems that the epidermis bordering the tumor site is more reliable, especially considering that the central epidermis covering the tumor mass is often thinned, ulcerated, or otherwise altered, making it difficult to examine, and it occasionally sloughs off during the staining procedure. GLI1 expression in both sites of the epidermal layer (border and central) is associated with GLI3 expression (*p* < 0.0001 for both locations), KRT16 expression (*p* < 0.0001 for both locations), and S100A7 expression (*p* < 0.0001 for both locations), as shown in Figure 6A–D, Supplementary Table S1, and Supplementary Figure S4. 

We show that staining of GLI1, KRT16, and S100A7 appears in the same places in the samples (same samples and different tumor slices) in all stages, while the localization of staining of GLI3 differs from the other proteins at lower stages (Supplementary Figure S2). For that reason, the analysis was further focused on the relationship between GLI1, KRT16, and S100A7. GLI1 shows a clear trend with all three tested proteins: a stronger staining of GLI1 corresponds to a stronger staining of GLI3 (*p* < 0.0001), KRT16 (*p* < 0.0001), and S100A7 (*p* < 0.0001) proteins. The same is true when comparing KRT16 and S100A7 expression (*p* < 0.0001) (Figure 6E–H). The data for other locations can be found in Supplementary Figure S5.

**Figure 7:**
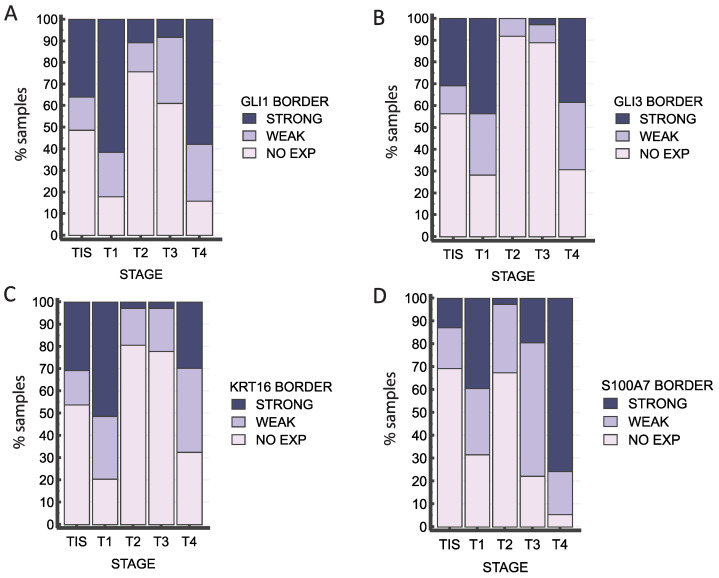
Distribution of intensities of staining for GLI1 (**A**), GLI3 (**B**), KRT16 (**C**), and S100A (**D**) in the epidermis bordering the tumor (BORDER) by stage (TIS, T1, T2, T3, and T4). All the stained proteins show significant differences between different stages (*p* < 0.0001 for all).

All the stained proteins (GLI1, GLI3, KRT16, and S100A7) show significant differences between different stages (*p* < 0.0001 for all), but only S100A7 also shows a statistically significant trend (post-hoc Jonckheere-Terpstra *p* < 0.0001), with higher stages showing more samples with strong staining (Figure 7 and Supplementary Table S1). The staining of all proteins demonstrated an unusual pattern of more intense staining at the lowest and highest stages, while stages T2 and T3 showed a reduction in the signal for all proteins (with the exception for S100A7 in T3). Surprisingly, even though regression shows the opposite pattern, with most occurrences of regression happening in the T2 stage, none of the staining intensities are associated with regression (not significant). 

### 2.5. Immune Infiltration Is an Important Feature in Melanoma Samples

**Figure 8:**
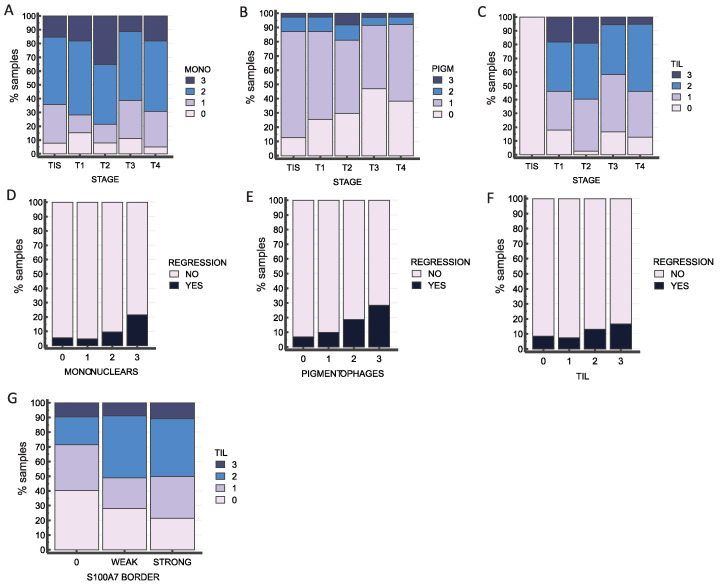
The role of immune infiltration in melanoma. Immune cells were determined as follows: none = 0, few = 1, medium/focally dense = 2, and dense = 3. Mononuclear cells are equally present in all stages (TIS, T1, T2, T3, and T4) (**A**) (*p* = 0.326), while pigmentophages (**B**) (*p* = 0.015) and TIL (**C**) (*p* < 0.0001) demonstrate a significant association with tumor stage. Mononuclear cells and pigmentophages are significantly associated with regression (*p* = 0.028 and *p* = 0.05, respectively) (**D**,**E**), and a similar trend is visible for TIL (**F**), but their value is not statistically significant (*p* = 0.238). (**G**) Association of S100A7 staining in the border epidermal layer and number of TIL (*p* = 0.013).

The involvement of immune cells is an important feature that is determined during the pathohistological examination of melanoma. Three types of immune cells are evaluated: mononuclear cells and pigmentophages at the borders, and tumor-infiltrating lymphocytes (TIL) within the tumor. Looking at the involvement of the three immune cell types across the stages, no significant differences were observed for the mononuclear cells (Figure 8A), but pigmentophages seem to become less abundant in higher tumor stages (*p* = 0.014), while TIL were significantly less abundant in TIS compared to other stages (*p* < 0.001) (Figure 8B–D). Mononuclear cells and pigmentophages are significantly associated with regression (*p* = 0.028 and *p* = 0.05, respectively), and a similar trend is visible for TIL, but their values are not statistically significant (*p* = 0.238). These features were also tested for association with our proteins of interest, but in the case of GLI proteins and KRT16, there was no statistical significance. The only protein showing some association with the number of TILs was S100A7 (*p* = 0.013), as shown in Figure 8G and Supplementary Table S1.

## 3. Discussion

In our previous study, we performed RNA-sequencing and ChIP-sequencing on melanoma cell lines in order to identify new HH-GLI-regulated targets [12]. qPCR validation of these results allowed us to narrow down the gene list to a few genes that show consistent and stable expression in the majority of the tested melanoma cell lines. Out of successfully validated targets of GLI proteins, we chose two of them for further validation presented in this study. These targets are S100A7 and KRT16, whose roles in skin diseases are already noticed but not completely studied and understood [32,36,37,38]. The cell lines were representative of the three most common mutations detected in melanoma: A375 harbors a mutation for BRAF, Mel224 a mutation for NRAS, and the CHL-1 line is wild type for these two genes.

Before using clinical melanoma samples, we successfully validated *KRT16* and *S100A7* as GLI targets on different in vitro models of melanoma cell lines, as their expression was strongly upregulated upon overexpression of all three GLI proteins. As a model for downregulated activity of the HH-GLI signaling pathway, we used melanoma cell lines resistant to GANT61, where both targets showed downregulated expression. We have also used a model for autocrine upregulation of the pathway—a cell line with a stable knock-in of SHH (CHL-1 SHH KI), where we show that SHH does not significantly affect expressions of *S100A7* and *KRT16*, although a slight upward expression trend is detected. Finally, to investigate the gene expression changes in 3D conditions of tumor cells, spheroids cultures from the A375 cell line were used. The data presented in Figure 1 strongly suggest that *S100A7* and *KRT16* expression are under the transcriptional control of GLI proteins in melanoma cell lines. So far, one previous study discovered *S100A7* as a gene whose transcription was synergistically induced by simultaneous activation of GLI1 and EGFR signaling. Using reporter assays, they showed enhanced induction of *S100A7* and *S100A9* but not of *PTCH1* activity by combinatorial GLI1 (GLI2*)/EGF signaling in keratinocytes [39].

In order to investigate the expression of GLI proteins and their targets in a more detailed manner, we obtained 190 FFPE melanoma samples for IHC analysis. Samples were collected between 2018 and 2022 and included melanoma in situ and all four T stages of melanoma. The age of patients ranged from 30 to 96 years. First, we analyzed the correlation between several clinicopathological characteristics (sex, existence of preexisting nevus, ulceration, and regression) in our samples. We have found a trend in the occurrence of melanoma within the preexisting nevus according to age, where nevus-associated melanoma is being diagnosed at a slightly younger age (Figure 2). These findings can possibly be explained by the behavioral patterns of patients, who are more likely to notice changes in the existing nevi than the appearance and changes in other locations (de novo melanoma). 

So far, a number of S100 family members have been found to be upregulated in many types of cancer (S100A1, S100A4, S100A6, S100A13, S100B, and S100P) [40]. Since its discovery in the 1980s, the expression of S100B has been used as a prognostic marker for malignant melanoma. In several studies from that period, S100B was found to be present at elevated levels in melanoma tumor biopsies but not in normal skin samples and non-melanoma tumors [41,42]. Regarding S100A7, two studies analyzing the GEO and TCGA databases demonstrated that it shows high expression in primary melanoma but decreased expression in metastatic melanoma [43,44]. Interestingly, one study pointed out that S100A7 can be detected in urine samples of melanoma patients and could potentially be used as a biomarker [45]. S100A7 was also found upregulated in tumors of the mucosa, such as cervical cancer [46], esophageal squamous cell carcinoma [47], or oral squamous cell carcinoma [48]. Our staining results of 190 samples show that expression of GLI1, GLI3, KRT16, and S100A7 was mostly detected in the epidermal layer overlying or bordering the tumor mass. The tumor mass itself was found positive for GLI1 and GLI3 in a proportion of samples, while S100A7 and KRT16 were rarely detected. It is interesting that we could not detect GLI2 in any of the samples, in the epidermal layer, or in the tumor itself. This is a possible limitation of our study, because we cannot confirm that the absence of GLI2 expression in clinical samples is not due to the choice of antibody used. Although we used GLI2 antibody from the same manufacturer and with the same IHC protocol in our past studies [49], this lot of antibody was discontinued and replaced with the one we are currently using in this study. However, for future studies, we would suggest using a different antibody than the one used to confirm that GLI2 expression is indeed not detectable. Another interesting finding is that GLI3 shows stronger staining in the basal layer in the epidermis of low-stage melanoma, unlike all other tested proteins. GLI3, unlike GLI1 and GLI2, has a dual role in HH-GLI signaling: it can act as both an activator and a repressor. It would be interesting to have different antibodies for the activator form of GLI3 and different antibodies for the repressor form, but this study was limited to the antibody that detects both forms. Finally, GLI1, GLI3, KRT16, and S100A7 were not detected in the healthy epidermis at the edges of the sample, suggesting that the staining is specific for the epidermis overlaying the tumor. One study from 1993 showed a similar situation in BCC. They noticed that all layers of the epidermis overlying the BCC showed more intense staining for the EGF receptor and keratin than the tumor masses. As one possible explanation for this observation, they suggest that BCC development is initiated by the proliferative properties of the epidermis [50]. Additionally, we suggest that the epidermis bordering the tumor site could be more reliable, especially considering that the central epidermis covering the tumor mass is often thinned, ulcerated, or otherwise altered, making it difficult to examine, and it occasionally sloughs off during the staining procedure. When comparing the expression of GLI1 to other target proteins in the same location (tumor vs. tumor, border vs. border, and central vs. central), there is generally a stronger correlation in the locations of the epidermal layer than the tumor mass.

We found a clear trend when considering the association between staining of GLI1 and other proteins: stronger staining of GLI1 corresponds to stronger staining of GLI3, KRT16, and S100A7 proteins. The same conclusion arises when comparing the expressions of KRT16 and S100A7. A stronger expression of KRT16 corresponds to a stronger expression of S100A7. A recent pan-cancer study identified KRT16 as one of the proteins with the highest correlation in expression with S100A7 [51]. Although all the stained proteins in our study show differences between different stages, only S100A7 also shows a statistically significant trend, with higher stages showing more samples with strong staining. 

It is not news that keratins are important in epidermal barrier maintenance, but there are also studies that associate keratins with innate immunity and inflammasome activity. The same studies also connect keratins to the S100 family of proteins [33,52]. The biology of melanoma is highly influenced by the interplay of melanoma cells with other cells within the tumor microenvironment, like immune cells [53], and by the production of numerous cytokines and chemokines that affect the tumor microenvironment [54]. This makes melanoma one of the most immunogenic tumors [55,56,57]. Tumor-infiltrating lymphocytes (TIL) are an important component of the tumor environment. More recently, it has been recognized that TIL signatures vary with cancer progression, which leads to investigating TIL as a potential prognostic biomarker [58]. Our results prove that immune infiltration is an important feature in clinical melanoma samples. Pigmentophages and TIL demonstrate a significant association with tumor stage, while mononuclear cells are equally present in all stages. We found mononuclear cells to be significantly associated with regression. Here, we could not prove a significant connection between KRT16 expression and immune cell infiltration. However, for S100A7, we found a correlation between the number of TILs and staining intensity. In previous studies, it was shown that S100A7 can protect the skin from microbial infection [21,59] and the involvement of S100A7 in the immune response has been demonstrated in several different models: in cultured keratinocytes, it stimulates IL-6 and IL-8 expression [60], while in esophageal squamous cell carcinoma, it promotes M2 macrophage infiltration and angiogenesis [47]. Other members of the S100 family, such as S100A12, have also been associated with a tumor inflammation signature [61]. Taken together, our IHC data support the results demonstrated in vitro that S100A7 and KRT16 expression correlate with the expression of GLI proteins (GLI1 and GLI3), although GLI2 was not detected. Surprisingly, this effect is far more pronounced in the epidermal layer of the tumor than in the tumor itself, suggesting that the interaction between the tumor and stromal compartments is relevant in the context of melanoma progression. We suggest further examination of KRT16 and S100A7, especially since S100A7 showed a statistically significant trend regarding tumor staging and staining intensity. 

## 4. Materials and Methods

### 4.1. Cell Culture

Human melanoma cell lines A375 (RRID: CVCL_0132), CHL-1 (RRID: CVCL_1122), and MEL224 (RRID: CVCL_U915) were kindly provided by Andreja Ambriović Ristov, PhD, and Neda Slade, PhD [12,62]. Cell lines A375 and CHL-1 were maintained in the recommended medium: Dulbecco’s Modified Eagle Medium (Merck KGaA, Darmstadt, Germany), while MEL224 was maintained in RPMI 1640 medium (Merck KGaA, Darmstadt, Germany), both supplemented with 10% FBS (Merck KGaA, Darmstadt, Germany), 1 mM sodium pyruvate, 1% streptomycin/penicillin, and 4 mM L-glutamine (Gibco Thermo Fisher Scientific, Waltham, MA, USA). RPMI was additionally supplemented with non-essential amino acids (Capricorn Scientific GmbH, Ebsdorfergrund, Germany).

### 4.2. Plasmids and Cell Transfection

For the GLI transfection experiments, cells were plated at a density of 3 × 10^5^ cells/well in 6-well plates, left to attach for 24 h, and then transfected with 5 μg of GLI expression plasmids: GLI1 (pcDNA4NLSMTGLI1, kindly gifted by Fritz Aberger, Ph.D.), GLI2 and GLI3 (p4TO6MTGLI2, pcDNA4/TO/GLI3richtig, both a kind gift from Milena Stevanović, PhD) using the Xfect reagent (Clontech, Mountain View, CA, USA) following the manufacturer’s instructions. A detailed protocol for the establishment and characterization of cell lines CHL-1 and MEL224 resistant to GANT-61 was published in [35]. In this publication, we used CHL-1 GANTR and MEL224 GANTR cell lines as models of the downregulated Hedgehog signaling pathway for additional validation of novel GLI targets [35].

### 4.3. Spheroid Culture

Spheroid cultures were established using the hanging drop method for three melanoma cell lines, namely, CHL-1, A375, and MEL224. Droplets with 25 µL of medium containing 2000 cells per droplet were seeded on the flat surface of a plastic Petri dish, which was then turned over. This method was already described in our previous study [63]. To prevent the drying of spheroid droplets over time, 5 mL of PBS buffer was added to the bottom of the Petri dish. The cells were allowed to form spheres for a maximum of 5 days. During these 5 days, they were collected for RNA isolation, measuring cell viability on a flow cytometer, and monitoring and photographing the shape and size of the formed spheres under a light BIB-100 Inverted Biological Microscope, (BOECO, Hamburg, Germany). 

### 4.4. Generation of the CHL1 SHH-KI Cell Line

A synthetic SHH gBlocks Gene Fragment (Integrated DNA Technologies, Coralville, IA, USA) was designed with the help of a publicly available tool (https://eu.idtdna.com/site/order/gblockentry, accessed on 22 August 2019). The nucleotide sequence of the SHH gene (NC_000007.14, accessed on 22 August 2019) was taken from the NCBI database, and restriction sites for EcoRI and NotI were added to the 5′ and 3′ ends, respectively. Synthetic gene fragment SHH was cloned into plasmid vector pAAVS1-Puro-DNR digested with NotI and EcoRI. The correct sequence of the inserted SHH gene fragment was verified using Sanger sequencing. The stable integration of the SHH gene in the genome of the CHL-1 cell line was performed using the CRISPR-Cas9 system with a commercial kit for gene insertion into the AAVS1 locus (GE100027, OriGene, Rockville, MA, USA). The transfection of the CHL-1 cell line was performed using the abovementioned protocol with Xfect reagent, but this time using 2.5 μg of pCas-Guide-AAVS1 and 2.5 μg of pAAVS1-Puro-DNR-SHH vectors. The following day, puromycin was added to the cells at a concentration of 1 μg/mL. The cells were left in the antibiotic for 15 days. Their medium with antibiotics was changed every second or third day. After antibiotic selection, the surviving cells were trypsinized, counted, and seeded in a 96-well plate at a density of 5 cells/mL in 100 μL of medium per well. This type of seeding ensures that there will usually be one cell in each well, which enables the development of a clonal line. Cells were maintained in a 96-well plate until they formed a single clear colony per well. Single colonies were screened for SHH gene expression, and the clonal line showing the strongest increase in SHH protein expression was used.

### 4.5. Quantitative PCR Analysis

Total RNA was extracted using TRIzol reagent (Invitrogen, Carlsbad, CA, USA) following the manufacturer’s instructions. cDNA was generated from 1 μg of RNA using the High-Capacity cDNA synthesis kit (Thermo Fisher Scientific, Waltham, MA, USA), and quantitative PCR analysis (qPCR) was performed on a CFX-96 instrument (Bio-Rad Laboratories, Hercules, CA, USA) using SsoAdvanced SYBR Green Supermix (Bio-Rad Laboratories, Hercules, CA, USA) with gene-specific primers. The cycling conditions were as follows: initial denaturation at 95 °C for 3 min; 40 cycles of 95 °C for 15 s, and 61 °C for 1 min; melting curve from 70 °C to 95 °C. The data were collected and analyzed using the CFX Manager Software v3.1 (Bio-Rad Laboratories, Hercules, CA, USA). Fold change was calculated relative to the RPLP0 housekeeping gene using the 2^−ΔΔCt^ method. Primer sequences for *GLI1*, *GLI2*, *GLI3*, *PTCH1*, *S100A7*, and *KRT16* used for qPCR were published before [12]. 

### 4.6. Patient Samples and Characteristics

Formalin-fixed paraffin-embedded (FFPE) melanoma tissues were collected from the archive of the Sestre Milosrdnice University Hospital Center, following the approval of the Ethical Committee (EP-18647/18-9). The collection included a total of 190 samples collected between 2018 and 2022: 39 tumors in situ (TIS), 39 T1, 37 T2, 36 T3, and 39 T4. The age of patients ranged from 30 to 96 years, with a median of 67 years. Clark and Breslow stages were determined where possible, but in the case of TIS, Breslow thickness and sometimes Clark scale are not determined if the tumor has not penetrated into the epidermis. In those cases, the values were presented as N/A. All other relevant clinicopathological characteristics are listed in Table 1.

### 4.7. Immunohistochemical Staining and Analysis

The slides were deparaffinized using Bioclear reagent (Biognost, Zagreb, Croatia), rehydrated in lowering concentrations of ethanol (100%, 90%, and 70%), and water. Boiling DAKO Retrieval Buffer (Agilent Technologies, Santa Clara, CA, USA) was used for 20 min for antigen retrieval, and the activity of endogenous peroxidase was blocked with 3% H_2_O_2_ in methanol for 10 min. Non-specific binding was blocked with DAKO Protein block solution (Agilent Technologies, Santa Clara, CA, USA), followed by primary antibody incubation in 2% BSA/TBST overnight at +4 °C in a humidified chamber. Primary antibodies were used at the following dilutions: GLI1 (Novus Biologicals, NB600-600) 1:100, GLI2 (sc-271786, Santa Cruz, Dallas, TX, USA) 1:100, GLI3 (GTX104362, Gene Tex, Irvine, CA, USA) 1:200, S100A7 (47C1068, Novus Biologicals, Centennial, CO, USA) 1:100, KRT16 (sc-53255, Santa Cruz, Dallas, TX, USA), 1:100. Detection and visualization were performed with the DAKO LSAB+ HRP kit and DAKO DAB chromogen (Agilent Technologies, Santa Clara, CA, USA), according to the manufacturers’ instructions. The slides were counterstained with hematoxylin (Biognost, Zagreb, Croatia), dehydrated with increasing concentrations of ethanol, washed with Bioclear solution (Biognost, Zagreb, Croatia), and fixed using Biomount resin (Biognost, Zagreb, Croatia). The slides were stained in batches of 40 slides per batch, and to avoid any possible batch effect, each batch contained samples from different tumor stages. For the negative control, slides were stained, omitting the primary antibody.

The slides were examined by two independent investigators, with a focus on the epidermis overlaying the tumor. The staining was scored at two locations for each sample: the epidermis directly above the tumor (marked CENTRAL in the subsequent analysis) and the epidermis at the border of the tumor mass and the surrounding epidermis (marked BORDER). The samples were scored in the following way: the staining intensity was scored as 0 (no signal), 1 (weak staining), 2 (moderate staining), and 3 (strong staining). The percentage of the stained cells was roughly divided into three categories: no positive cells, <50% of positive cells, and >50% positive cells [64]. By combining those two measurements, the samples were divided into three categories: negative (no staining), weak (all samples with intensity 1 + samples with <50% cells with intensity 2), and strong (samples with >50% cells with intensity 2 and all samples with intensity 3).

### 4.8. Statistical Analysis

For qPCR experiments, an independent sample *t*-test was used to infer the differences in gene expression. Clinical data and protein staining intensities were analyzed with the Chi-square test (including the Chi-squared test for trend) and the Mann-Whitney or Kruskal–Wallis test (including the Jonckheere-Terpstra test for trend), depending on the type of data and number of groups. The data were analyzed using MedCalc for Windows V.19.4.1 (MedCalc Software Ltd., Ostend, Belgium). Two-tailed *p*-values less than 0.05 were considered statistically significant.

## 5. Conclusions

We present here for the first time that S100A7 and KRT16 are transcriptional targets of GLI proteins in melanoma. They are detected in melanoma patient samples in the same structures as the GLI1 protein and show differences in expression between melanoma stages. S100A7 additionally shows an association with immune infiltration. Most interestingly, in the patient samples, all proteins are localized in the epidermis overlaying the tumor and not necessarily within the tumor mass. We suggest that staining of the epidermis should be taken into consideration when examining melanoma samples and hope that our study could lead to investigating S100A7 as a potential biomarker in melanoma.

## Figures and Tables

**Table 1:** Baseline clinicopathological characteristics of melanoma samples grouped by stage. Presented is the number of samples (n).

Characteristic	TIS	T1	T2	T3	T4	Total
Sex						
m	19 (17.4%)	26 (23.9%)	18 (16.5%)	19 (17.4%)	27 (24.8%)	109 (100%)
f	20 (24.7%)	13 (16.0%)	19 (23.5%)	17 (21.0%)	12 (14.8%)	81 (100%)
Clark						
I	39 (95.1%)	2 (4.9%)	0 (0.0%)	0 (0.0%)	0 (0.0%)	41 (100%)
II	0 (0.0%)	27 (100%)	0 (0.0%)	0 (0.0%)	0 (0.0%)	27 (100%)
III	0 (0.0%)	9 (20.5%)	25 (56.8%)	7 (15.9%)	3 (6.8%)	44 (100%)
IV	0 (0.0%)	1 (1.4%)	12 (16.7%)	28 (38.9%)	31 (43.1%)	72 (100%)
V	0 (0.0%)	0 (0.0%)	0 (0.0%)	1 (20.0%)	4 (80%)	5 (100%)
Breslow						
N/A	39 (97.5%)	1 (2.5%)	0 (0.0%)	0 (0.0%)	0 (0.0%)	40 (100%)
I	0 (0.0%)	34 (100%)	0 (0.0%)	0 (0.0%)	0 (0.0%)	34 (100%)
II	0 (0.0%)	4 (12.9%)	27 (87.1%)	0 (0.0%)	0 (0.0%)	31 (100%)
III	0 (0.0%)	0 (0.0%)	10 (22.7%)	33 (75%)	1 (2.3%)	44 (100%)
IV	0 (0.0%)	0 (0.0%)	0 (0.0%)	3 (7.7%)	36 (92.3%)	39 (100%)
V	0 (0.0%)	0 (0.0%)	0 (0.0%)	0 (0.0%)	1 (100%)	1 (100%)
Preexisting nevus						
Yes	11 (35.5%)	8 (25.8%)	6 (19.4%)	4 (12.9%)	2 (6.5%)	31 (100%)
No	28 (17.6%)	31 (19.5%)	31 (19.5%)	32 (20.1%)	37 (23.3%)	159 (100%)
Regression						
Yes	3 (15.0%)	6 (30.0%)	7 (35.0%)	2 (10.0%)	2 (10.0%)	20 (100%)
No	36 (21.2%)	33 (19.4%)	30 (17.6%)	34 (20.0%)	37 (21.8%)	170 (100%)
Ulceration						
Yes	0 (0.0%)	1 (1.8%)	5 (8.9%)	19 (33.9%)	31 (55.4%)	56 (100%)
No	39 (29.1%)	38 (28.4.%)	32 (23.9%)	17 (12.7%)	8 (6.0%)	134 (100%)
Mononuclear cells						
None	3 (15.8%)	6 (31.6%)	3 (15.8%)	4 (21.1%)	2 (10.5%)	19 (100%)
Few	11 (27.5%)	5 (12.5%)	5 (12.5%)	10 (25.0%)	10 (25.0%)	40 (100%)
Medium dense	19 (20.2%)	21 (22.3%)	16 (17.0%)	18 (19.1%)	20 (21.3%)	94 (100%)
Dense	6 (16.2%)	7 (18.9%)	13 (35.1%)	4 (10.8%)	7 (18.9%)	37 (100%)
Pigmentophages						
None	5 (8.6%)	10 (17.2%)	11 (19.0%)	17 (29.3%)	15 (25.9%)	58 (100%)
Few	29 (26.6%)	24 (22.0%)	19 (17.4%)	16 (14.7%)	21 (19.3%)	109 (100%)
Medium/focal	4 (25.0%)	4 (25.0%)	4 (25.0%)	2 (12.5%)	2 (12.5%)	16 (100%)
Dense	1 (14.3%)	1 (14.3%)	3 (42.9%)	1 (14.3%)	1 (14.3%)	7 (100%)
TIL						
None	39 (68.4%)	7 (12.3%)	1 (1.8%)	6 (10.5%)	5 (8.8%)	57 (100%)
Few	0 (0.0%)	11 (20.4%)	14 (25.9%)	15 (27.8%)	13 (24.1%)	54 (100%)
Medium/focal	0 (0.0%)	14 (23.0%)	15 (24.6%)	13 (21.3%)	19 (31.1%)	61 (100%)
Dense	0 (0.0%)	7 (38.9%)	7 (38.9%)	2 (11.1%)	2 (11.1%)	18 (100%)

N/A—not available; TIL—tumor-infiltrating lymphocytes.

## Data Availability

Data is contained within the article and Supplementary Materials.

## Supplementary Materials

The following supporting information can be downloaded at: https://www.mdpi.com/article/10.3390/ijms25116084/s1.
