# Supplementary material for: GLI Transcriptional Targets S100A7 and KRT16 Show Upregulated Expression Patterns in Epidermis Overlying the Tumor Mass in Melanoma Samples
Source: Int J Mol Sci. 2024 May 31;25(11):6084. doi: 10.3390/ijms25116084 (PMC11172526; doi:10.3390/ijms25116084)
Supplement: Supplementary file 1 [file ijms-25-06084-s001.zip › Supplementary figures.pdf]

Supplementary information

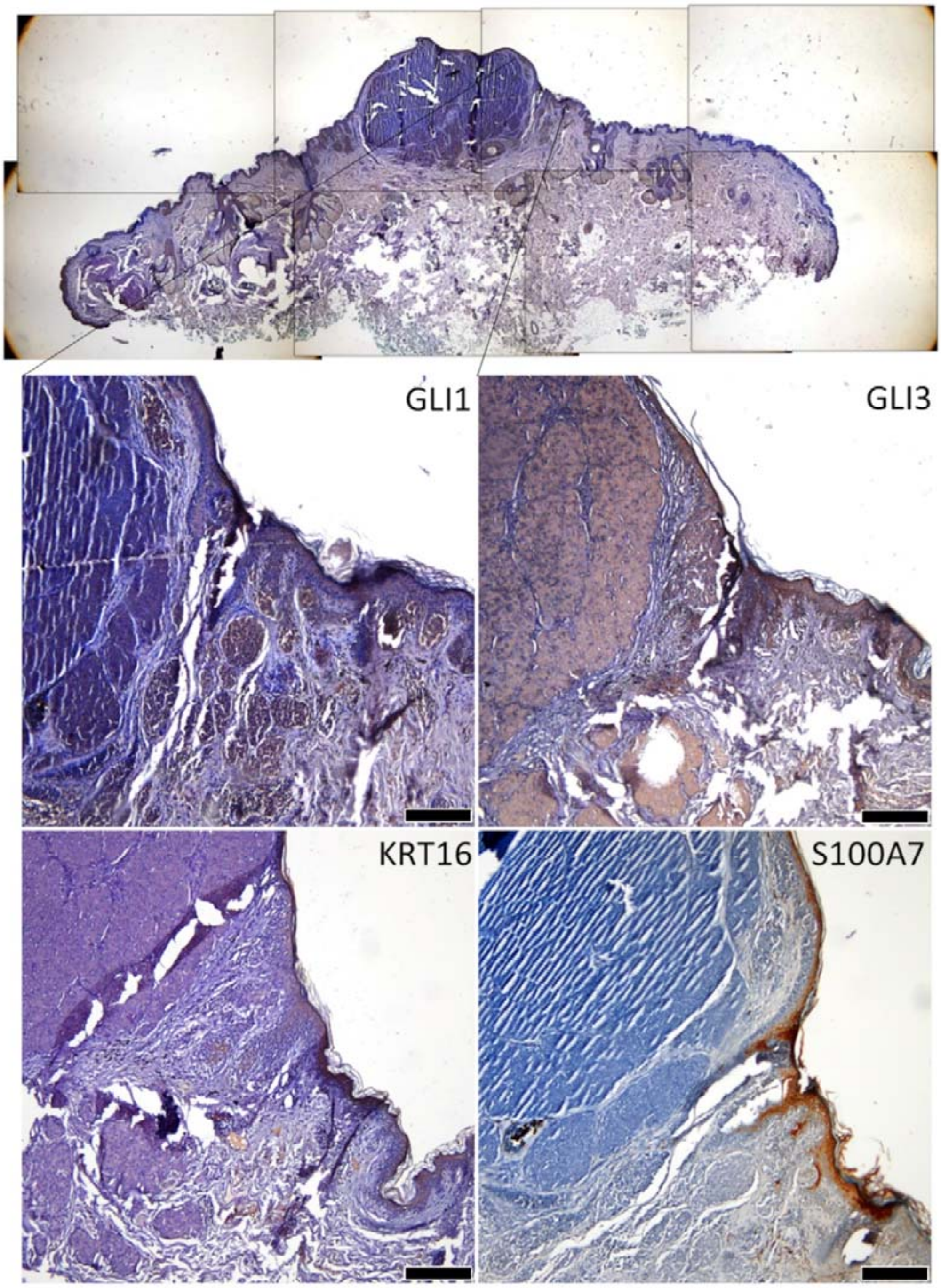

Supplementary figure S1. Comparison of staining of four tested proteins (GLI1, GLI3, S100A7, KRT16) on a slide with all three investigated areas: tumor tissue, border epidermis and epidermis overlaying the tumor. All four proteins show staining in both the central epidermis overlaying the tumor mass and the border epidermis, while GLI1 and GLI3 additionally show staining of the tumor mass. Scale bar = 200 µm.

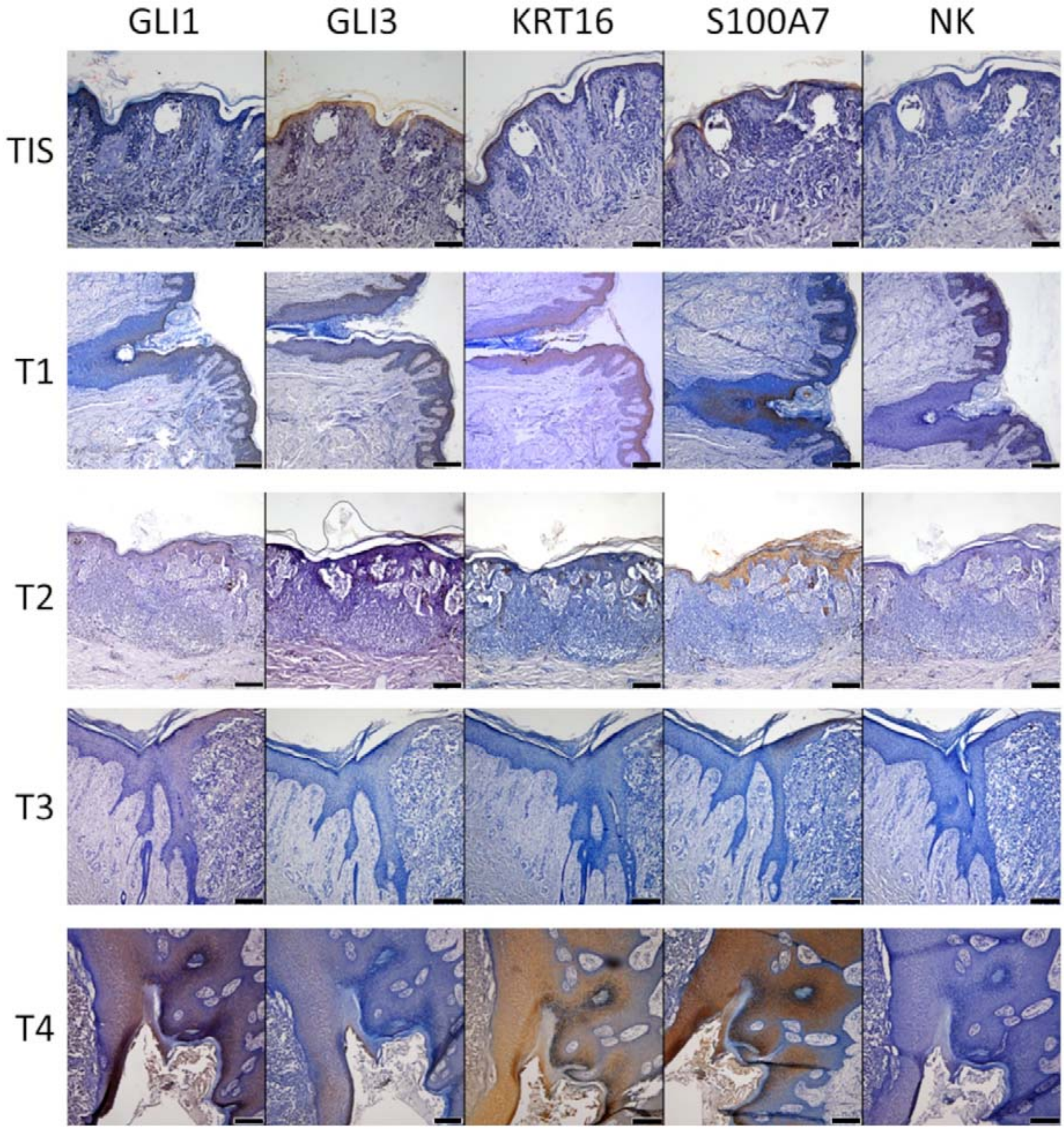

Supplementary figure S2. Examples of staining for each stage of melanoma and each tested protein (GLI1, GLI3, KRT16, S100A7 and negative control). NC denotes negative control. Scale bar = 200 µm.

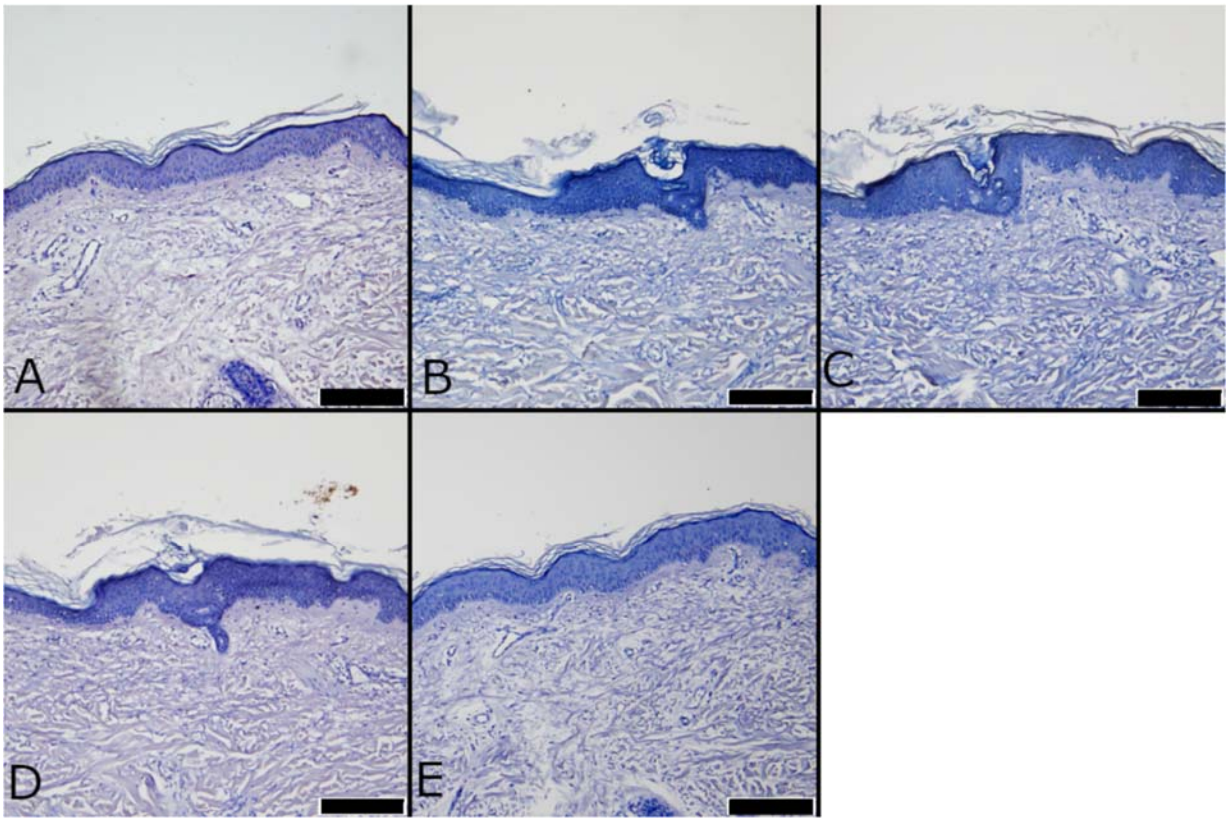

Supplementary figure S3. Representative staining of GLI1 (A), GLI3 (B), KRT16 (C) and S100A7 (D) in healthy epidermis adjacent to the tumor. Panel E represents no primary antibody control stain. Scale bar = 200 µm.

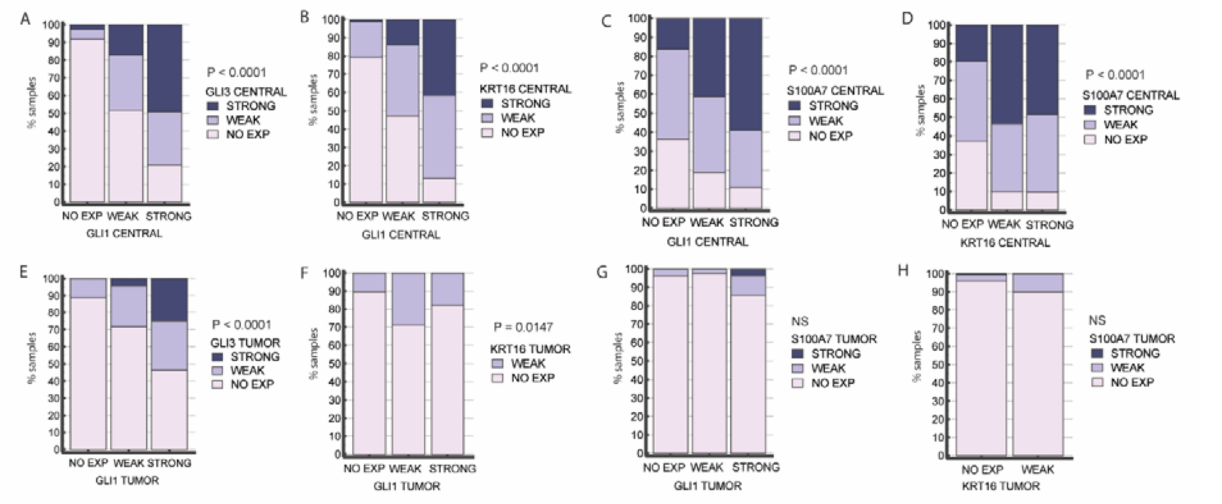

Supplementary figure S4. Protein expression of GLI1 (A), GLI3 (B), KRT16 (C) and S100A7 (D) in the central epidermis (CENTRAL) and epidermis bordering the tumor (BORDER). There is a significant association between protein expression on both sites (border epidermis and central epidermis), P<0.0001 for all proteins.

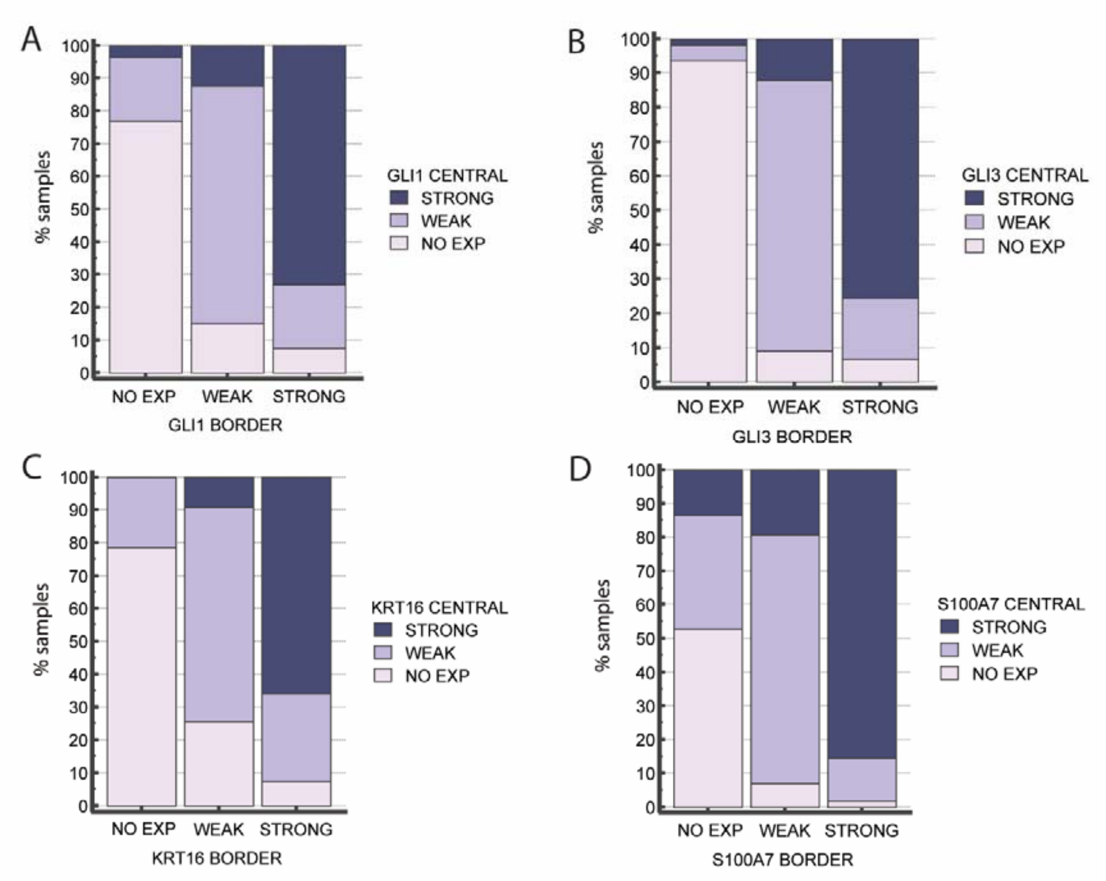

Supplementary figure S5. Comparison of protein expression (GLI1 *vs* GLI3, KRT16 and S100A7 and KRT16 *vs* S100A7) in central epidermis overlying the tumor (A-D) and in tumor mass (E-H). There is a significant association between GLI1 and GLI3 expressions (E), (P<0.0001), and between GLI1 and KRT16 (F), (P=0.015).
